# Supplementary material for: The effect of tempi and mode on the rating of the perceived emotion in music
Source: PLoS One. 2026 May 18;21(5):e0348069. doi: 10.1371/journal.pone.0348069 (PMC13183231; doi:10.1371/journal.pone.0348069)
Supplement: S1 File — (DOCX) [file pone.0348069.s001.docx]

Supporting Information

S1 File. Supporting information.

Access to musical and control stimuli

All stimuli are available at:

https://figshare.com/projects/Music_and_control_stimuli/246239

Færøvik, Ulvhild (2025). Noise stimuli (brown, pink, and white). figshare. Media. https://doi.org/10.6084/m9.figshare.28855481.v1

Færøvik, Ulvhild (2025). Song 1 major. figshare. Media. https://doi.org/10.6084/m9.figshare.28854632.v1

Færøvik, Ulvhild (2025). Song 1 minor. figshare. Media. https://doi.org/10.6084/m9.figshare.28854659.v1

Færøvik, Ulvhild (2025). Song 2 major. figshare. Media. https://doi.org/10.6084/m9.figshare.28854662.v1

Færøvik, Ulvhild (2025). Song 2 minor. figshare. Media. https://doi.org/10.6084/m9.figshare.28854665.v1

Færøvik, Ulvhild (2025). Song 3 major. figshare. Media. https://doi.org/10.6084/m9.figshare.28854671.v1

Færøvik, Ulvhild (2025). Song 3 minor. figshare. Media. https://doi.org/10.6084/m9.figshare.28854674.v1

Færøvik, Ulvhild (2025). Song 4 minor. figshare. Media. https://doi.org/10.6084/m9.figshare.28855454.v1

Færøvik, Ulvhild (2025). Song 4 major. figshare. Media. https://doi.org/10.6084/m9.figshare.28855460.v1

Færøvik, Ulvhild (2025). Song 5 major. figshare. Media. https://doi.org/10.6084/m9.figshare.28855463.v1

Færøvik, Ulvhild (2025). Song 5 minor. figshare. Media. https://doi.org/10.6084/m9.figshare.28855469.v1

Færøvik, Ulvhild (2025). Voice recordings. figshare. Media. https://doi.org/10.6084/m9.figshare.28855484.v1

Figures


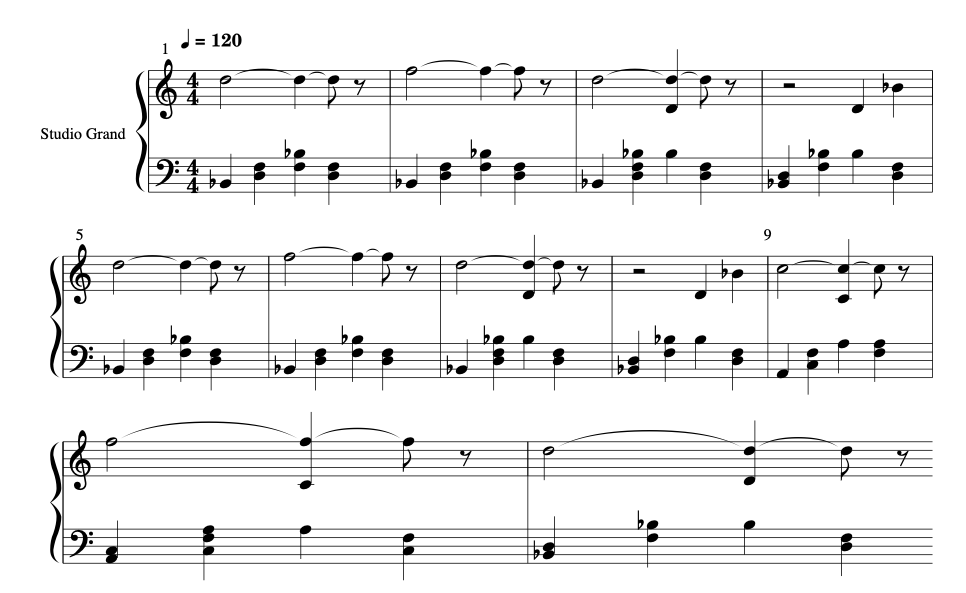
 Figure A1. Sheet music for Stimulus 1 (major mode).

Note: The full piece is longer than 21 seconds. Displayed here is the notation for 120 beats per minute.

Figure A2. Sheet music for Stimulus 1 (minor mode).


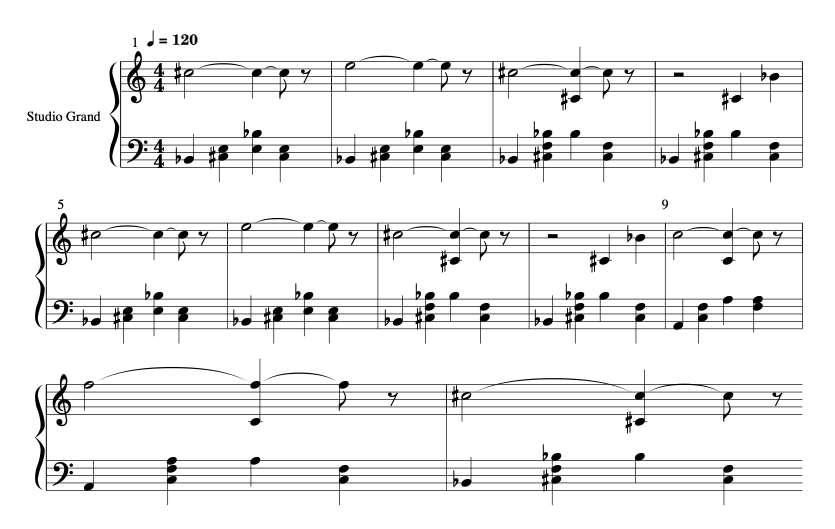
 Note: The full piece is longer than 21 seconds. Displayed here is the notation for 120 beats per minute.


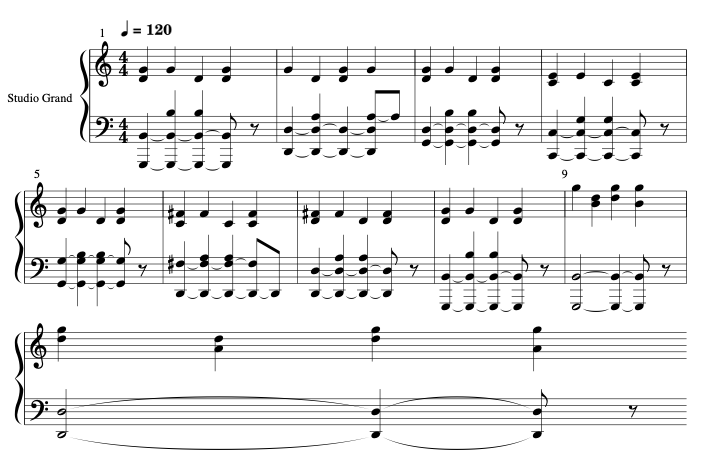
 Figure A3. Sheet music for Stimulus 2 (major mode).

Note: The full piece is longer than 21 seconds. Displayed here is the notation for 120 beats per minute.


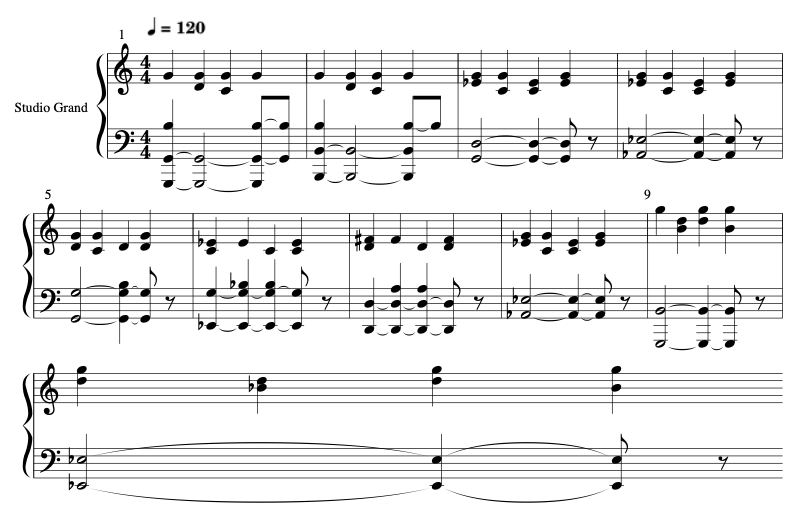
 Figure A4. Sheet music for Stimulus 2 (minor mode).

Note: The full piece is longer than 21 seconds. Displayed here is the notation for 120 beats per minute.


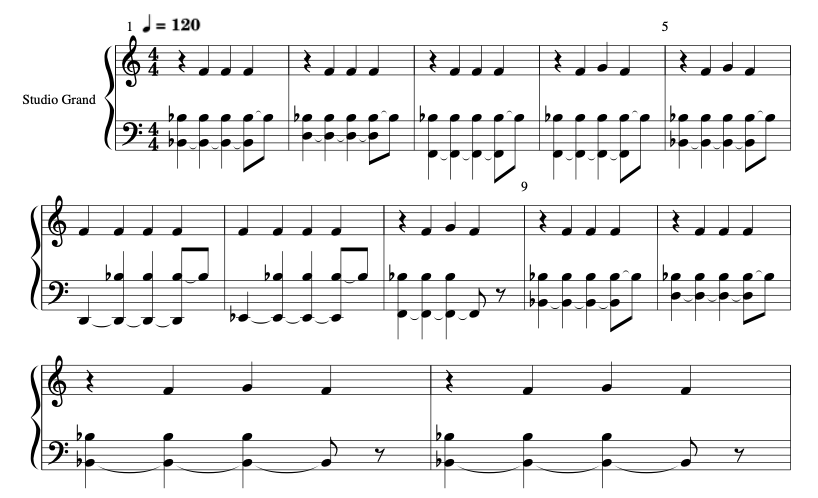
 Figure A5. Sheet music for Stimulus 3 (major mode).

Note: The full piece is exactly 21 seconds. Displayed here is the notation for 120 beats per minute.

Figure A6. Sheet music for Stimulus 3 (minor mode).


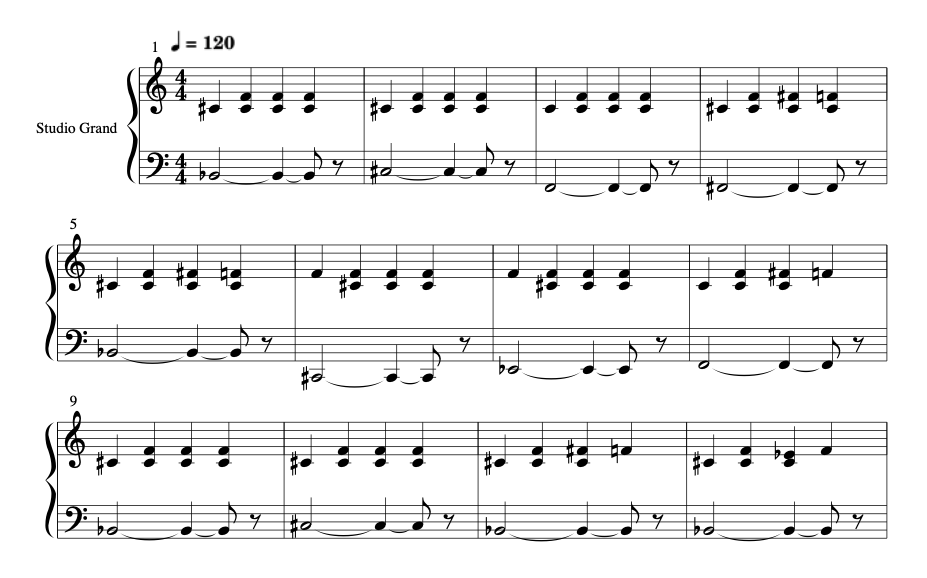
 Note: The full piece is exactly 21 seconds. Displayed here is the notation for 120 beats per minute.


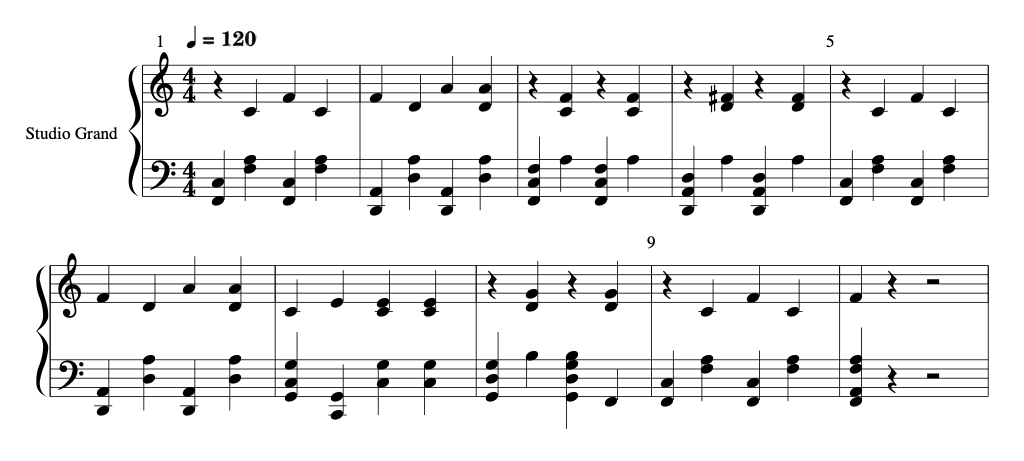
 Figure A7. Sheet music for Stimulus 4 (major mode).

Note: The full piece is 18 seconds long, so it was repeated at the faster tempi. Displayed here is the notation for 120 beats per minute.


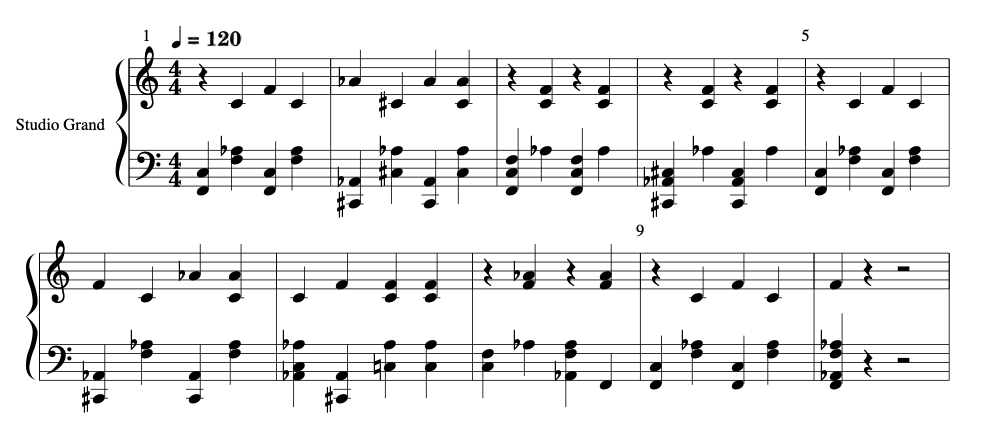
 Figure A8. Sheet music for Stimulus 4 (minor mode).

Note: The full piece is 18 seconds long, so it was repeated at the faster tempi. Displayed here is the notation for 120 beats per minute.

Figure A9. Sheet music for Stimulus 5 (major mode).


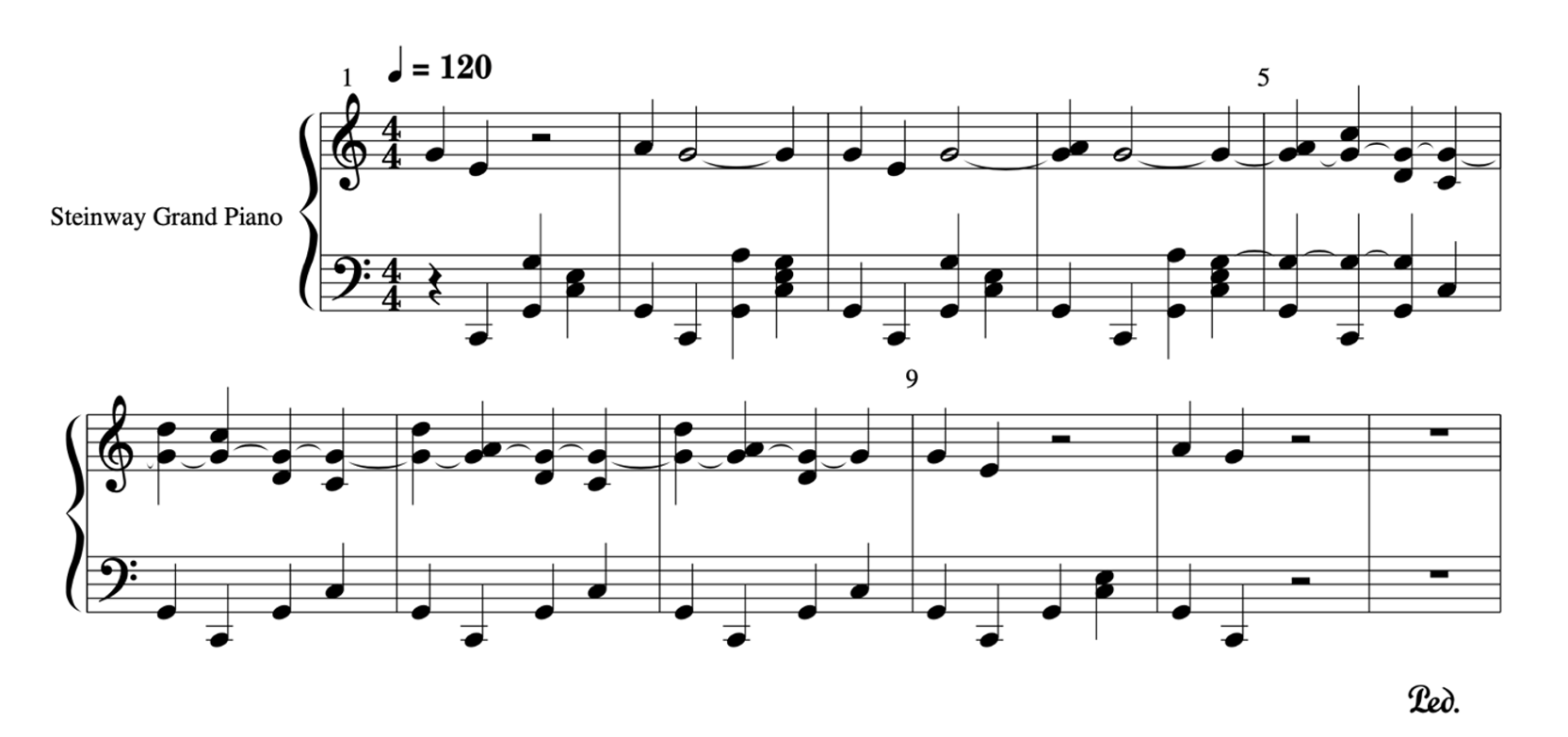


Note: The full piece consists of 10 bars (shown here). For the 60 BPM version, only the first 6 bars were presented at 21 seconds, while all 10 bars were included at 120 BPM.


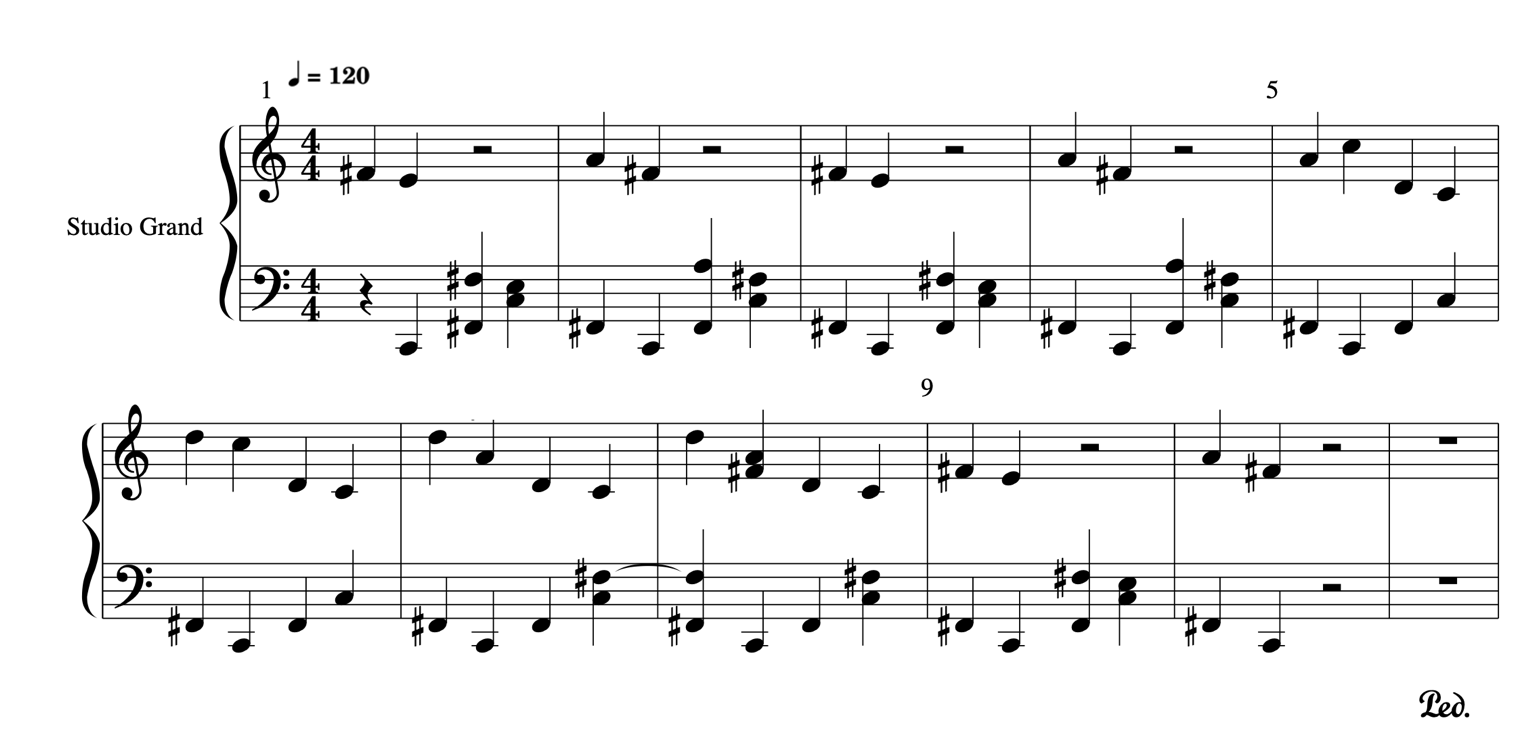
 Figure A10. Sheet music for Stimulus 5 (minor mode).

Note: The full piece consists of 10 bars (shown here). For the 60 BPM version, only the first 6 bars were presented at 21 seconds, while all 10 bars were included at 120 BPM.

Tables

Table A1. Norwegian and English translations of the GEMS-9 scale with additional synonyms.

| 2nd order factors | 1st order factors Norwegian GEMS-9 | 1st order factors English GEMS-9 | Additional synonyms (Norwegian) | Additional synonyms (English) |
| --- | --- | --- | --- | --- |
| Sublim | Undring | Wonder | Lykke, forbløffet, forlokket, beveget | Happiness, amazed, enticed, moved |
|  | Overskridelse | Transcendence | Inspirert, følelse av åndelighet, frysninger, følelse av overskridelse | Inspired, sense of spirituality, chills, feeling of transcendence |
|  | Ømhet | Tenderness | Forelsket, kjærlig, sensuell, øm, oppmyket | In love, loving, sensual, tender, softened |
|  | Nostalgi | Nostalgia | Sentimental, drømmende, nostalgisk, melankolsk | Sentimental, dreamy, nostalgic, melancholic |
|  | Fredfylt | Peacefulness | Rolig, avslappet, indre ro, beroliget, meditativ | Calm, relaxed, inner peace, reassured, meditative |
| Vital | Energisk | Power | Energisk, triumferende, flammende, sterk, heroisk | Energetic, triumphant, blazing, strong, heroic |
|  | Glede | Joyful activation | Stimulerende, glad, animert, dansende, underholdt | Stimulating, happy, animated, dancing, entertained |
| Uroende | Anspent | Tension | Oppildnet, nervøs, anspent, utålmodig, irritert | Aroused, nervous, tense, impatient, irritated |
|  | Sørgmodig | Sadness | Trist, sorgfull | Sad, sorrowful |

Table A2. Sublimity ratings across the five compositions (major mode).

| Composition | 60 BPM Mean (SD) | 100 BPM Mean (SD) | 120 BPM Mean (SD) | 150 BPM Mean (SD) |
| --- | --- | --- | --- | --- |
| Composition 1 | 4.09 (1.79) | 3.56 (1.62) | 3.54 (1.65) | 3.10 (1.60) |
| Composition 2 | 3.76 (1.66) | 3.29 (1.54) | 3.04 (1.57) | 2.47 (1.40) |
| Composition 3 | 4.05 (1.74) | 3.62 (1.65) | 3.32 (1.55) | 2.85 (1.54) |
| Composition 4 | 3.69 (1.66) | 3.13 (1.55) | 2.83 (1.45) | 2.40 (1.35) |
| Composition 5 | 3.76 (1.75) | 3.23 (1.58) | 3.02 (1.56) | 2.62 (1.45) |

Table A3. Unease ratings across the five compositions (major mode).

| Composition | 60 BPM Mean (SD) | 100 BPM Mean (SD) | 120 BPM Mean (SD) | 150 BPM Mean (SD) |
| --- | --- | --- | --- | --- |
| Composition 1 | 2.26 (1.51) | 2.03 (1.32) | 1.87 (1.26) | 1.77 (1.17) |
| Composition 2 | 2.41 (1.56) | 2.05 (1.32) | 1.92 (1.24) | 1.82 (1.20) |
| Composition 3 | 2.65 (1.67) | 2.43 (1.51) | 2.27 (1.45) | 2.13 (1.37) |
| Composition 4 | 3.10 (1.66) | 2.91 (1.56) | 2.68 (1.46) | 2.48 (1.41) |
| Composition 5 | 2.43 (1.57) | 2.42 (1.49) | 2.23 (1.41) | 2.15 (1.40) |

Table A4. Vitality ratings across the five compositions (major mode).

| Composition | 60 BPM Mean (SD) | 100 BPM Mean (SD) | 120 BPM Mean (SD) | 150 BPM Mean (SD) |
| --- | --- | --- | --- | --- |
| Composition 1 | 1.96 (1.16) | 2.84 (1.45) | 3.49 (1.57) | 4.34 (1.53) |
| Composition 2 | 2.45 (1.47) | 3.08 (1.46) | 3.85 (1.47) | 4.76 (1.41) |
| Composition 3 | 2.03 (1.26) | 2.52 (1.35) | 3.26 (1.53) | 4.28 (1.51) |
| Composition 4 | 2.18 (1.34) | 2.85 (1.46) | 3.59 (1.52) | 4.41 (1.49) |
| Composition 5 | 1.86 (1.11) | 2.79 (1.40) | 3.46 (1.48) | 4.23 (1.55) |

Table A5. Sublimity ratings across the five compositions (minor mode).

| Composition | 60 BPM Mean (SD) | 100 BPM Mean (SD) | 120 BPM Mean (SD) | 150 BPM Mean (SD) |
| --- | --- | --- | --- | --- |
| Composition 1 | 3.42 (1.70) | 3.11 (1.59) | 2.94 (1.55) | 2.67 (1.50) |
| Composition 2 | 3.82 (1.66) | 3.14 (1.52) | 3.05 (1.50) | 2.75 (1.45) |
| Composition 3 | 4.09 (1.71) | 3.85 (1.63) | 3.62 (1.66) | 3.24 (1.63) |
| Composition 4 | 3.53 (1.71) | 3.08 (1.54) | 2.91 (1.54) | 2.49 (1.47) |
| Composition 5 | 3.24 (1.68) | 2.90 (1.62) | 2.67 (1.53) | 2.57 (1.63) |

Table A6. Unease ratings across the five compositions (minor mode).

| Composition | 60 BPM Mean (SD) | 100 BPM Mean (SD) | 120 BPM Mean (SD) | 150 BPM Mean (SD) |
| --- | --- | --- | --- | --- |
| Composition 1 | 4.46 (1.70) | 4.35 (1.70) | 4.32 (1.65) | 4.37 (1.68) |
| Composition 2 | 3.48 (1.61) | 3.60 (1.59) | 3.57 (1.57) | 3.52 (1.56) |
| Composition 3 | 3.67 (1.80) | 3.49 (1.66) | 3.38 (1.66) | 3.24 (1.57) |
| Composition 4 | 4.14 (1.74) | 3.84 (1.58) | 3.84 (1.58) | 3.80 (1.66) |
| Composition 5 | 4.14 (1.69) | 4.33 (1.65) | 4.22 (1.59) | 4.03 (1.80) |

Table A7. Vitality ratings across the five compositions (minor mode).

| Composition | 60 BPM Mean (SD) | 100 BPM Mean (SD) | 120 BPM Mean (SD) | 150 BPM Mean (SD) |
| --- | --- | --- | --- | --- |
| Composition 1 | 1.64 (1.04) | 1.91 (1.11) | 2.28 (1.24) | 2.95 (1.48) |
| Composition 2 | 2.40 (1.47) | 2.91 (1.53) | 3.46 (1.52) | 3.94 (1.50) |
| Composition 3 | 1.81 (1.12) | 2.24 (1.28) | 2.90 (1.49) | 3.54 (1.59) |
| Composition 4 | 2.16 (1.43) | 2.82 (1.56) | 3.11 (1.57) | 3.97 (1.66) |
| Composition 5 | 1.65 (1.01) | 2.00 (1.14) | 2.47 (1.36) | 2.63 (1.52) |

Table A8. Repeated-measures ANOVA results for sublimity ratings.

| Effect | Wilks’ Λ | F | df (Hyp) | df (Error) | p | ηp² |
| --- | --- | --- | --- | --- | --- | --- |
| Tempi | .98 | 6.00 | 3 | 1035 | < .001 | .01 |
| Mode | .98 | 18.32 | 1 | 1037 | < .001 | .01 |
| Tempi × Mode | .98 | 4.40 | 3 | 1035 | .004 | .01 |
| Mode × Age | .98 | 18.18 | 1 | 1037 | < .001 | .01 |
| Mode × Musicianship | .99 | 6.75 | 1 | 1037 | .009 | .00 |
| Tempi × Mode × Age | .99 | 2.84 | 3 | 1035 | .037 | .00 |
| Tempi × Mode × Gender | .98 | 5.40 | 3 | 1035 | < .001 | .01 |

Table A9. Mean and standard deviation of sublimity ratings.

| Tempi | Mode | N | Mean | SD |
| --- | --- | --- | --- | --- |
| 60 | Major  Minor | 1042 | 3.8  3.6 | 1.3  1.2 |
| 100 | Major  Minor | 1042 | 3.3  3.2 | 1.1  1.1 |
| 120 | Major  Minor | 1042 | 3.1  3 | 1.1  1 |
| 150 | Major  Minor | 1042 | 2.7  2.7 | 1  1 |

Table A10. Repeated-measures ANOVA results for unease ratings.

| Effect | Wilks’ Λ | F | df (Hyp) | df (Error) | p | ηp² |
| --- | --- | --- | --- | --- | --- | --- |
| Tempi | .97 | 9.90 | 3 | 1034 | < .001 | .02 |
| Mode | .84 | 189.51 | 1 | 1036 | < .001 | .15 |
| Tempi × Mode | .98 | 4.40 | 3 | 1035 | .004 | .01 |
| Tempi × Age | .98 | 6.50 | 3 | 1037 | < .001 | .01 |
| Tempi × Gender | .97 | 6.50 | 3 | 1034 | < .001 | .02 |
| Tempi × Musicianship | .99 | 3.00 | 3 | 1034 | .029 | .00 |
| Mode × Age | .85 | 179.57 | 3 | 1036 | < .001 | .14 |
| Mode × Gender | .99 | 8.00 | 1 | 1036 | .005 | .00 |
| Mode × Musicianship | .97 | 28.84 | 1 | 1036 | < .001 | .02 |
| Tempi × Mode × Age | .98 | 4.40 | 3 | 1034 | .004 | .01 |

Table A11. Mean and standard deviation of unease ratings.

| Tempi | Mode | N | Mean | SD |
| --- | --- | --- | --- | --- |
| 60 | Major  Minor | 1041 | 2.5  3.9 | 1.1  1.2 |
| 100 | Major  Minor | 1041 | 2.3  3.7 | 1  1.1 |
| 120 | Major  Minor | 1041 | 2.2  3.8 | 1  1.1 |
| 150 | Major  Minor | 1041 | 2  3.8 | 0.9  1.1 |

Table A12. Repeated-measures ANOVA results for vitality ratings.

| Effect | Wilks’ Λ | F | df (Hyp) | df (Error) | p | ηp² |
| --- | --- | --- | --- | --- | --- | --- |
| Tempi | .90 | 35.10 | 3 | 1035 | < .001 | .09 |
| Mode | .88 | 135.86 | 1 | 1037 | < .001 | .11 |
| Tempi × Mode | .94 | 19.10 | 3 | 1035 | < .001 | .05 |
| Tempi × Gender | .97 | 7.70 | 3 | 1035 | < .001 | .02 |
| Mode × Education | .99 | 7.90 | 1 | 1037 | .005 | .00 |
| Mode × Gender | .97 | 26.40 | 1 | 1037 | < .001 | .02 |
| Mode × Musicianship | .99 | 4.10 | 1 | 1037 | .004 | .00 |
| Tempi × Mode × Age | .98 | 5.90 | 3 | 1035 | < .001 | .01 |
| Tempi × Mode × Gender | .98 | 5.30 | 3 | 1035 | .001 | .01 |

Table A13. Means and standard deviations of vitality ratings.

| Tempi | Mode | N | Mean | SD |
| --- | --- | --- | --- | --- |
| 60 | Major  Minor | 1042 | 2  1.9 | .8  .8 |
| 100 | Major  Minor | 1042 | 2.7  2.3 | 1  .9 |
| 120 | Major  Minor | 1042 | 3.5  2.8 | 1  1 |
| 150 | Major  Minor | 1042 | 4.4  3.4 | 1.1  1.1 |

Table A14. Repeated-measures ANOVA results for age differences and post-hoc comparisons.

| Stimuli | Age | N | Mean | SD | F | DF | Sig. | Eta squared |
| --- | --- | --- | --- | --- | --- | --- | --- | --- |
| Sublime_60_major | 18-35  36-59  60 + | 543  353  148 | 4.03  3.77  3.60 | 1.23  1.32  1.38 | 8.313 | 2 | <.001 | .01 |
| Sublime_100_major | 18-35  36-59  60 + | 647  425  181 | 3.54  3.22  3.10 | 1.14  1.18  1.17 | 15.405 | 2 | <.001 | .02 |
| Sublime_120_major | 18-35  36-59  60 + | 647  425  181 | 3.28  3.02  3.15 | 1.14  1.14  1.16 | 8.490 | 2 | <.001 | .01 |
| Sublime_150_major | 18-35  36-59  60 + | 646  424  181 | 2.80  2.56  2.56 | 1.09  1.09  1.09 | 9.163 | 2 | <.001 | .01 |
| Vital_120_major | 18-35  36-59  60 + | 647  425  181 | 3.63  3.47  3.30 | 1.08  1.12  1.00 | 1.020 | 2 | <.001 | .01 |
| Vital_150_major | 18-35  36-59  60 + | 646  424  181 | 4.54  4.33  4.16 | 1.13  1.20  1.12 | 2.506 | 2 |  | .01 |
| Vital_150_minor | 18-35  36-59  60 + | 644  422  180 | 3.29  3.50  3.55 | 1.12  1.11  1.07 | 7.669 | 2 |  | .00 |
| Unease_100_Major | 18-35  36-59  60 + | 647  425  181 | 2.3  2.33  2.63 | 1.00  1.04  1.12 | 7.023 | 2 | <.001 | .01 |
| Unease_120_Major | 18-35  36-59  60 + | 647  425  181 | 2.15  2.14  2.45 | .95  .97  1.09 | 7.590 | 2 | <.001 | .01 |
| Unease_60_minor | 18-35  36-59  60 + | 644  422  181 | 4.19  3.85  3.59 | 1.14  1.31  1.34 | 20.668 | 2 | <.001 | .03 |
| Unease_100_minor | 18-35  36-59  60 + | 644  422  181 | 3.97  3.56  3.39 | 1.05  1.20  1.28 | 26.118 | 2 | <.001 | .04 |
| Unease_120_minor | 18-35  36-59  60 + | 644  422  180 | 4.17  3.63  3.38 | 1.05  1.19  1.20 | 49.457 | 2 | <.001 | .07 |
| Unease_150_minor | 18-35  36-59  60 + | 644  422  180 | 4.13  3.51  3.28 | 1.05  1.20  1.16 | 61.657 | 2 | <.001 | .09 |

Table A15. T-tests for control stimuli.

| Stimuli | N | Mean | SD | df | T | Sig. two tailed | Eta square |
| --- | --- | --- | --- | --- | --- | --- | --- |
| Sublimity major-  Sublimity control | 1066 | 3.2  1.9 | 1  .9 | 1065 | 37.28 | <.001 | .5 |
| Sublimity minor-  Sublimity control | 1267 | 3.1  1.9 | .9  .9 | 1266 | 36.12 | <.001 | .5 |
| Unease major  Unease control | 1065 | 2.3  3 | .9  1.3 | 1064 | -16.74 | <.001 | .2 |
| Unease minor-  Unease control | 1267 | 3.8  2.9 | 1  1.3 | 1266 | 20.25 | <.001 | .2 |
| Vitality major-  Vitality control | 1066 | 3.1  1.8 | .8  .9 | 1065 | 43.55 | <.001 | .6 |
| Vitality minor-  Vitality control | 1267 | 2.6  1.8 | .8  .9 | 1266 | 29.68 | <.001 | .4 |
