# Supplementary material for: The effect of tempi and mode on the rating of the perceived emotion in music
Source: PLoS One. 2026 May 18;21(5):e0348069. doi: 10.1371/journal.pone.0348069 (PMC13183231; doi:10.1371/journal.pone.0348069)
Supplement: Attachment — Submitted filename: Response to reviewers.docx [file pone.0348069.s003.docx]

Dear editor,

We thank you and the reviewers for the thoughtful and constructive feedback on our manuscript, titled The Effect of Tempo and Mode on the Rating of the Perceived Emotion in Music. We have revised the manuscript thoroughly in response to the comments and believe the changes have substantially improved its clarity, structure, and contribution to the field. Below, we address each point raised by the reviewers, with our responses following each comment in **bold**.

**Reviewer #1**

**Overall this is an interesting study but the manuscript needs a bit more work, I think. I have a small question about the statistical analysis, hence my response to the prior question. I see the authors have promised to include the data upon publication. My comments are bellow.**

We thank the reviewer for this encouraging overall assessment and for their valuable suggestions. We have addressed each point in detail below. **The authors present an interesting study that develops on existing foundations in emotion perception in music. They acknowledge that this is a partial replication, delving into familiar ground but with some advantages such as having a large sample size. Their method deviates somewhat from prior studies in their choice of ratings terminology, utilising bespoke compositions, and different control stimuli. While there are clear advantages to this, I feel the authors could do more to contextualise their research and emphasise their contribution to the field. The writing can be a bit vague and disjointed, and certain theoretical concepts are not very well explained. The results could be presented in a clearer and more concise way to help the reader grasp the findings. I think there is clearly a valuable source of data here, and the article can be improved with more consideration to its presentation and contextualisation in the research field. More details as follows:

Introduction
In your discussion of different theories of emotion, I think there may be some confusion between notions of discrete and dimensional models of emotion and emotivist and cognitivist theories of emotions, or at least the descriptions and distinctions of different concepts (how we conceptualise emotions in music and how we measure emotions in music) are not very clear. You may want to review this. You might also consider delving more into constructionist theories and their implications as posited by Cespedes-Guevara & Eerola, who you cite. See also Lennie & Eerola, 2022.**

We revised the Introduction to clearly distinguish between discrete and dimensional models of emotion and included a new discussion on constructionist perspectives, citing Cespedes-Guevara & Eerola (2018) and Lennie & Eerola (2022). We now explain how these models relate to GEMS-9 and clarify the conceptual foundation of the study. **In your discussion of different measures, you point out that Eerola & Vuoskoski found that a dimensional model of valence, energy, and tension outperformed other models, including the GEMS-9. I think you can better explain why you still chose to use the GEMS-9 scale: why not adopt valence (sublime), energy (vital), and tension (uneasy)? Did you reflect on two-dimensional models of affect (valence and arousal)? One point you make is that the GEMS scales are designed for measuring either evoked (felt?) or perceived emotion – is that not the case for other scales?**

We now clearly articulate the rationale for selecting GEMS-9 second-order factors. This includes their focus on domain-specific aesthetic emotions and the flexibility to compare with both dimensional and discrete approaches. We also cite Pearce & Halpern (2015) and Zentner et al. (2008) to support this choice and clarify that other scales do not all support both perceived and felt emotion as GEMS-9 does.

**Method
It would be nice to know a bit more about the compositions. I appreciate they may have been used in other studies and those articles may have more details, but a summary here, if only brief, would be helpful as well to give a better sense of the intentions/design, including a consideration of any other musical factors that might contribute and/or need controlling, such as dynamics, rhythm, and timbre. For example, I note having listened to some of the samples through the Dropbox link that they were MIDI piano tracks with constant crotchet beat rhythms. This could be important for considering the research against other studies with different kinds of stimuli.**

We have expanded the Methods section to describe the compositions in more detail, including the use of a MIDI piano with a constant crotchet rhythm, and our rationale for this choice. We also note that the stimuli were pilot-tested to confirm beat perception consistency.

**I think there needs to be a bit more detail on the choice of ratings questions, as mentioned above. I would clarify the ratings scale and details of how the measure is presented in the Materials section, not in the Procedure as it is currently, to make it clear and concise. There is also mention of additional synonyms being included; what were they? In the supplementary material I can see the nine primary factors, and it strikes me that there are five for sublime but only two for the others. Was anything considered to balance this out? Was this the reason for the additional synonyms?**

We moved the GEMS-9 scale description to the Materials section and clarified the synonyms used (now also listed in the supplementary materials). We address the asymmetry in the number of terms, especially for the Sublime factor, and reflect on how this may relate to the valence biases in music-related vocabulary.

**Analysis
If I have understood correctly, you have aggregated the ratings of individual compositions by tempo and mode groupings. Did you test if there was any effect of composition? I can see the justification for this, but it would be good to see it clearly explained in the text. This relates to the question about knowing the details of the compositions and being able to interpret or consider these aspects of the methodology.**

We did not test for individual composition effects, as our design aggregated ratings by tempo and mode to strengthen generalizability. We now explain this choice and rationale more explicitly in the analysis section.

**Results
I think you can make the results section a bit clearer by perhaps summarising the numerical outputs/ANOVA results in a table. It is a bit unwieldly in its current form. You have included similar tables in the supplementary documents. I also wonder if you have sufficiently corrected for multiple testing – you include a correction in your t-tests for the control conditions, did you consider applying any corrections across your other tests?**

We restructured the Results section, added a comprehensive ANOVA summary table, and clarified the use of Bonferroni correction. Effect sizes are also reported throughout, and all adjustments are now mentioned in the Analysis section.

**Discussion
The first three paragraphs are a repetition of the results and shouldn’t be necessary in a Discussion section.

I would be interested to hear more about the advantages of your study (e.g., large sample size) and where you believe it has especially contributed to the field. You acknowledge early in the paper that this is something of a replication and you compare your results against previous work; what are the new implications of your study? Why are your results important? Are there any methodological recommendations you can make?**

We revised the opening of the Discussion to avoid repeating results and rewrote sections to highlight the unique contributions of the study — including the large sample size, controlled stimuli, second-order GEMS factors, and a strong methodological framework. We also articulate the theoretical implications more clearly.

**Limitations
You mention some limitations of the GEMS scale factors, and you acknowledge in your Discussion that there are challenges related to variations of emotion terms. I think you can acknowledge this further. The last paragraph of your Limitations section describes being able to compare with dimensional ratings, but while the three categories you’ve chosen may be considered relevant to valence (sublime?), energy (vitality) and tension (uneasy), for example, the specific terms may have different connotations for your participants. You have tried to control this with the addition of other terms and synonyms in the presentation of the ratings, but it could still be a limiting factor. Are there other ways your choice of terms could be a strength?**

We have expanded the Limitations section to reflect more critically on the influence of translating GEMS-9 terms into Norwegian. We also argue that using less familiar, nuanced words may have encouraged thoughtful reflection (i.e., System 2 processing), which could be seen as a strength in this context. **As mentioned previously, your choice of music is another important factor in this study. What are the strengths and limitations of the stimuli selection? What about ecological validity, perhaps?**

We now explicitly acknowledge that while ecological validity is limited by the use of simplified stimuli, this choice allowed us to isolate the effects of tempo and mode without other musical confounds. **Small points
Presentation of figures, tables and graphs – could be tidied up a bit to make things more presentable, for example removing the _ in the labels/text. Consider recommended formatting styles e.g., APA. Graphs could be more distinguished with better labelling or grouping – if you’ve labelled the bars, I don’t think you need to include a legend, or vice versa. You might want to consider other ways of identifying/grouping variables to make it clearer – figures 1-3 could show bars grouped by tempo, colour coded by mode, for example.**

We revised Figures 1–3 for better visual clarity, grouping by tempo and color-coding by mode. Labels were cleaned up, legends clarified, and the overall style now conforms more closely to APA standards. **Some typographical errors throughout, for example in the Introduction; ‘Genova’ instead of ‘Geneva’; Results section: ‘See table 1. For mean and standard deviations. See figure 1. For visual representation.’ – capitalisation following the full stop after each number; missing ‘p’ when reporting the p value in most of the stat reporting. Generally, the article could be revised for clarity and precision in the writing.**

These have been corrected throughout the manuscript.

**Reviewer #2**

**This is a paper of moderate relevance. Though the paper provides a lot of data with a huge number of participants (N = 1280), there are not many new ideas that may trigger the interest of the reader. The statistical analysis seems to be sound but what is missing to some extent is a broadening of scope and some background explanation and broader positioning of the findings. The findings are not really innovative, and it can be questioned what is really new: finding that tempo and mode influence the ratings of music is quite common knowledge. A more critical elaboration and interpretation of the findings would make the paper stronger.**

We thank Reviewer 2 for their thoughtful critique and useful suggestions. We address each point in turn below. **General remarks

• The major strength of the paper is the huge number of participants.
• The coherence of the collected data from the literature review is not totally convincing.**

We have expanded the Introduction and Discussion to more clearly position the findings in the broader literature, including constructionist, evolutionary, and aesthetic perspectives. We also emphasize the value of replication and methodologically rigorous studies, especially with large samples.

**• Some terms are used in a rather loose way and should be defined more strictly. E.g., what is meant with “uneasy music”? Focal concepts such as sublimity, unease, and vitality should also be explained more in detail.**

These terms are now more clearly defined in relation to their underlying GEMS-9 first-order factors. The introduction has been revised to present these categories with more precision and theoretical grounding.

**• The statistics must be explained somewhat more in detail. How are the values for the ratings computed?**

We added an explanation of how Mean scores were calculated — specifically, averages across five stimuli per tempo/mode condition — in both the Methods and Results sections. **• The reference list is substantial but more original sources could be mentioned, especially with regard to the distinction between discrete and dimensional approach to emotions. This holds in particular for the work of Scherer.**

We have added several references to Scherer’s work (2004, 2008, 2013) to support our discussion of aesthetic emotions and the Component Process Model (CPM).

**• Try to avoid references of papers that are submitted but not yet in press.**

Unfortunately, this is unavoidable; our other paper is still under review, but we’ve made the citation more transparent. Taking it out would mean duplicating and making this paper longer.

**• The most important take home message of the paper is not very strong and does not add very much to already existing knowledge.**

The Conclusion has been revised to clearly articulate the main contributions and why they matter — including the role of structure (tempo and mode) in shaping perceived musical emotion, even under tightly controlled conditions.

**• In its current form the paper seems to focus more on effects sizes rather than on critical elaboration of the findings. Some more in-depth discussions of the findings should make the paper stronger.**

We agree that effect sizes are important but have now added more interpretation and commentary in the Discussion to complement the statistical perspective. Effect sizes emphasise whether the findings are believable or not; they lower the chance of random occurrences. This paper emphasises methodology, which is why the discussion follows methodological approaches.

**• Stronger motivations could be given for some methodological choices, such as, e.g., the use of second-order factors.**

We now provide a clearer rationale for using second-order factors, including their interpretability and fit with previous studies that used GEMS-9 at multiple levels.

**• The conclusion is very short. A stronger take home message should be given. What are the major findings? What is new? What is different from current knowledge?**

We revised and lengthened the Conclusion to reflect key findings, their contribution to existing knowledge, and future directions.

**Detailed comments
• Page 9, last sentence: this sentence seems to be grammatically incomplete. Please reword.
• Page 10, 2nd paragraph: Please add additional basic references for the description of discrete emotion as applied to music (Eerola; Reybrouck, and others, see suggestions below)
• Page 10, last par.: reference should be made also to the so-called “aesthetic emotions”. Reference should be made to Scherer. There is a comma lacking after (GEMS-9). Please explain somewhat more in detail what is meant with a domain-specific model.
• Page 11, 1st par.: Please explain somewhat more in detail the meaning of the second-order factors and their relation to the first-order factors.
• Page 11, 3rd par.: please substitute “valence” for “valance”. This holds also for all other appearances.
• Page 13: hypotheses: the 3rd hypothesis is very generalizing; the concept of “unease” must be better explained.
• Page 13, penultimate par.: the categories of uneasy, sublime and vital must be much better specified.
• Page 14, 1st par.: it should be explained more clearly that for each of the three neutral voice recordings there was one male and one female speaker**.  **• Page 14: it is not easy to identify the uploaded music examples: the used abbreviations to tag the examples should be explained somewhat more in detail. For example, what is the meaning of B_N, P_N, SUBLIM, UROLIG, V-1, etc. Please try to be as clear as possible to avoid seeking efforts by the readers.
• Page 15, 1st. par.: The target was set at 1562. Please explain shortly why this number was chosen and what was the rationale behind. This can be very short but try to be intuitive.
• Page 17, results: this is a very abrupt beginning of presenting the results. Please insert a short initial text that announces that the three variables (sublime, uneasy, vital) will be described.
• Page 17, table 1. It is not directly clear how the values (ratings) for the mean have been computed. Please explain as clearly as possible.
• Page 18, Figure 1. Is it possible to insert the significance level in the figure y using *, **, ***?
• Page 25, 2nd par.: there seems to be a contradicting assertion here: an increase in both unpretentious and sophisticated music. Or these categories not opposed to each other?**

**Suggestions for additional rerences (not mandatory)
Eerola, T. & Vuoskoski, J. (2013) A Review of Music and Emotion Studies: Approaches, Emotion Models, and Stimuli. Music Perception, 30(3), 307–340. DOI: 10.1525/MP.2012.30.3.307
Eerola, T., Vuoskoski, J., Peltola, H.-R., Putkinen, V., Schäfer, K. (2018). An integrative review of the enjoyment of sadness associated with music. Physics of Life Reviews 25, 100–121.** [**https://doi.org/10.1016/j.plrev.2017.11.016**](https://doi.org/10.1016/j.plrev.2017.11.016) **Lamont, A. & Eerola, T. (2011). Music and emotion: Themes and development. Musicae Scientiae,15 (2), 139-145.
Reybrouck, M. & Eerola, T. (2017). Music and its inductive power: a psychobiological and evolutionary approach to musical emotions. Frontiers in Psychology, 8, Art.No. 494. Open Access
Robinson, J. (2009). Aesthetic emotions (philosophical perspectives). In D. Sander & K. R. Scherer (Eds.), The Oxford companion to emotion and the affective sciences (pp. 6–9). New York: Oxford University Press.
Scherer, K. (2008). Music evoked emotions are different – more often aesthetic than utilitarian. Behavioral and Brain Sciences, 31, 5, 595.**

- Grammar and typographical errors have been corrected.
- Additional references on discrete emotions, aesthetic emotions, and domain-specific models have been added.
- Definitions and structure of GEMS-9 second-order categories are now clearer.
- Stimuli files have been renamed for clarity and are documented with DOIs.
- The participant number rationale is clarified in the Method section.
- Results are introduced more smoothly.
- Significance levels are reported in-text; we chose not to add asterisks to figures to avoid clutter.
- Regarding unpretentious vs. sophisticated: we note these are not mutually exclusive preferences, and listeners may endorse both.
- We added most of the suggested literature.

Once again, we thank the reviewers and editorial team for their time and input, which we believe have helped improve the manuscript. We hope the revised version meets your expectations and look forward to your decision.

Sincerely,
Ulvhild Færøvik [Corresponding Author]
On behalf of all authors
