# Supplementary material for: The effect of tempi and mode on the rating of the perceived emotion in music
Source: PLoS One. 2026 May 18;21(5):e0348069. doi: 10.1371/journal.pone.0348069 (PMC13183231; doi:10.1371/journal.pone.0348069)
Supplement: Attachment — Submitted filename: Response_to_reviewers_auresp_2.docx [file pone.0348069.s004.docx]

Thank you to all the reviewers for the hard work and thorough comments. We have made the changes you asked for. In this letter, we detail each change to the separate reviewers.

**Reviewer #1**

We thank Reviewer #1 for their careful reading of the revised manuscript and for their constructive and encouraging feedback. We are pleased that the reviewer finds the methodological revisions satisfactory and appreciates the added detail regarding stimuli and measures. Below, we respond to the remaining points raised.

**Conceptual clarification of emotion models and theories**

We are grateful to the reviewer for highlighting the need for clearer conceptual distinctions between discrete versus dimensional emotion models, felt versus perceived emotions, and underlying emotion theories (e.g., emotivist, cognitivist, constructionist). We agree that these distinctions were not sufficiently explicit in the previous version.

To address this, we have revised the Introduction to clearly differentiate between:
(a) emotion theories (how emotions are generated and conceptualised),
(b) emotion measurement frameworks (discrete vs. dimensional models), and
(c) the phenomenological target of measurement (felt vs. perceived emotion).

We have added an explicit clarifying paragraph early in the Introduction stating that discrete and dimensional models are measurement frameworks that can be applied to both felt and perceived emotions, and that the felt–perceived distinction primarily concerns task instructions rather than the choice of emotion model itself. In addition, we have revised wording that could be read as implying that dimensional models cannot be applied to felt emotions, as this was not our intended claim.

**Positioning of the GEMS-9 and use of second-order factors**

Relatedly, we have clarified our use of the GEMS-9 framework. While we employ the second-order factors of the GEMS-9, which resemble dimensional emotion spaces, we now explicitly state that we retain the domain-specific, music-focused conceptualisation of aesthetic emotions rather than treating the scale as a generic dimensional model. This clarification is now made explicit in the Introduction and Methods sections to avoid conceptual ambiguity.

**Strengthening the contribution and value of the study**

We appreciate the reviewer’s suggestion to strengthen the final paragraph of the Introduction by more clearly justifying the value of the present study. We have substantially revised this section to more explicitly frame the study as a high-powered, methodologically rigorous replication and extension of prior work on tempo and mode. In particular, we now emphasise the importance of replication for establishing robustness and generalizability in music–emotion research, especially given the variability of stimuli, emotion models, and sample sizes in the existing literature. This revised framing better situates the study’s contribution without engaging in an extended discussion of the replication crisis.

**Clarity, proofreading, and consistency**

Finally, we have conducted a thorough proofreading of the manuscript. This included correcting typographical errors, improving clarity in several passages, standardising terminology (e.g., consistent use of “first-order” and “second-order”), and addressing formatting inconsistencies noted by the reviewer.

We again thank Reviewer #1 for their thoughtful feedback, which has helped us improve the conceptual clarity, positioning, and overall quality of the manuscript.

**Reviewer #2**

We sincerely thank Reviewer #2 for the careful and detailed evaluation of our manuscript. We appreciate the critical feedback and have revised the manuscript substantially to improve clarity, methodological transparency, and theoretical positioning. Below, we respond to each of the reviewer’s general and detailed comments.

**1. The contents are of moderate relevance with a lot of repetition of known facts. The major question is: what is new?**

We appreciate this important question and have clarified the novelty of the study in the Introduction. Specifically, we now emphasise that the current study is a conceptual replication study. The novelty lies in combining a large sample size, multiple structurally matched compositions, second-order GEMS and direct comparison with non-musical control stimuli.

**2. The research questions are very general, which makes them not really suitable for an empirical study.**

We have reformulated and specified the research questions to clearly indicate that they concern graded effects of tempo and mode within the present stimulus set. We now avoid overly broad claims about “music” as a general category. And clarify that we examine parameter-level effects rather than holistic compositional effects.

**3. The style of writing is not very mature (young scholars?) and the academic standards are not very high.**

We have carefully revised the manuscript for clarity, structure, and academic tone. In particular, the Results section has been rewritten to reduce SPSS-style reporting. Effect sizes are now explicitly interpreted (small, medium, large). Redundant passages have been removed. Paragraph structure has been tightened throughout.

We hope the revised version reflects a clearer and more mature academic style.

**4. The style of referencing is rather weak. Some major references are lacking, there are also multiple references to submitted papers that are not yet accepted.**

We have removed references to “submitted” manuscripts wherever possible. Where necessary, such work has either been replaced with published sources or clearly identified as preprints.

We have also strengthened the theoretical background with additional references in sections discussing dimensional versus categorical models and second-order emotional constructs.

**5. The research questions are very general and reductionistic. More motivation is needed to explain why some selections have been made: why only tempo and mode? What about other parameters (dynamics, agogics, timbre, etc.)?**

We have clarified the rationale for focusing on tempo and mode, specifically as the pilot study included dynamics (the referenced preprint). Both parameters are among the most consistently studied structural determinants of musical emotion. They can be systematically manipulated while holding other parameters constant. The aim of the study was not to model the full complexity of music, but to examine controlled parameter-level effects.

We explicitly acknowledge in the revised manuscript that emotional responses to music are multi-determined and that tempo and mode represent experimentally isolatable contributors rather than exhaustive determinants.

**6. The methodology seems to have some potential, but the methods themselves are very badly explained. Figures and tables are introduced in the text without the needed accompanying explaining text, which makes it very difficult to interpret them. Many questions can also be raised about the collection of the data, which have been collected in less standardized circumstances.**

The Methods and Analysis sections have been substantially revised to improve transparency. Specifically we now provide a step-by-step description of how ratings were aggregated across compositions within tempo–mode conditions. The rationale for aggregation is explained. The statistical model is described clearly, including within- and between-subject factors. Interpretation of Wilks’ lambda and partial eta squared is clarified. Effect size benchmarks are explicitly provided.

We hope these revisions address the concern regarding methodological clarity.

**7. There is overall too much generalization. For example, speaking of music as a general category is very problematic to be consider as an independent variable. There are so many kinds of music, all with a different potential effect on listeners.**

We agree that music is not a homogeneous category. We have revised the manuscript to clarify that conclusions apply to the present stimulus set, the manipulated structural parameters and the operationalized emotional dimensions.

Claims have been carefully delimited.

**8. The paper as whole seems to be an example of method-driven analysis, with a lot of statistical processing and computation, but with a scarcitiy of starting ideas and challenging intuitions. The theoretical background is also rather weak.**

We have revised the Introduction to strengthen the theoretical framing and clarify the conceptual motivation behind the analyses. The Results section has also been restructured to emphasize interpretive coherence rather than statistical detail.

Additionally, we have added a synoptic summary table (Table 1) that highlights the overall pattern of effects across emotional dimensions, making the theoretical structure of findings more transparent.

**Detailed Comments**

**Dimensional vs categorical models (p. 10)**

We have expanded this section and added additional references to clarify the distinction and empirical support for dimensional approaches.

**Dimensional models outperforming (p. 10)**

The relevant paragraph has been revised to avoid overly strong claims and to provide clearer references supporting the statement.

**Second-order categories (p. 11–13)**

We have expanded the explanation of the second-order dimensions (sublime, uneasy, vital), including conceptual definitions, operationalisation in the GEMS framework and clarification that participants received standardised descriptors. We also included the intercorrelations among the first- and second-order factors in GEMS.

We also discuss potential interpretational variability in terms such as “sublime.”

**“Valence” correction**

Corrected throughout.

**Strong claim (p. 12)**

The statement has been moderated and additional references added.

**Reductionism and isolation of parameters (p. 13)**

We have strengthened the rationale for isolating tempo and mode and explicitly acknowledge the limitations of parameter-level approaches.

**Submitted references**

Removed or replaced as noted above.

**Synoptic table and effect sizes (p. 17)**

In response to this important suggestion, we have added a synoptic summary table (Table 1) presenting main effects and key interactions across all three emotional dimensions. Reported F-values, degrees of freedom, p-values, and partial eta squared consistently. Explicitly stated effect size benchmarks in the Analysis section. Clarified interpretation of Wilks’ lambda. Clarified how mean values were computed.

Full statistical tables are retained in supplementary materials for transparency.

**Text in Discussion is better suited for Introduction (p. 24)**

We have relocated the indicated paragraphs to the Introduction to improve structural coherence.

**Figures (pp. 32 ff.)**

All figures have been revised to include clearer captions and expanded explanatory text in the Results section to guide interpretation.

**Concluding Remark**

We are grateful for the reviewer’s detailed critique. The manuscript has undergone substantial revision to improve clarity, methodological transparency, theoretical positioning, and presentation of results. While the empirical findings remain unchanged, their structure and interpretation are now presented more clearly and concisely.

We hope the revised manuscript addresses the reviewer’s concerns and demonstrates the contribution of the study more convincingly.

**Reviewer #3**

Thank you for reviewing our manuscript. We appreciate the hard work and your comments.

**1. Rephrase or substantiate the statement that tempo and mode are “inconsistently examined across emotion models.”** Either specify the precise inconsistencies the paper addresses (e.g., differing stimuli, measures, or theoretical mappings) or soften the claim to position the work as a large-scale, stimulus-controlled extension of established findings. The manuscript itself cites many prior tempo/mode studies.

Thank you for this suggestion. We have rephrased this statement to avoid overgeneralization. Rather than implying broad inconsistency, we now position the study as a large-scale, stimulus-controlled extension of established findings, while acknowledging the substantial body of prior research on tempo and mode.

**2) In the introduction, I recommend streamlining the sections on emotion models to improve logic:** briefly introduce discrete, dimensional, and domain-specific approaches, then clearly justify the use of GEMS-9. The current text reads as somewhat disjointed.

We appreciate this comment. The previous version of the introduction had expanded in response to earlier reviewer requests for additional theoretical detail, which may have affected its coherence. We have now substantially streamlined the section by briefly introducing discrete, dimensional, and domain-specific approaches in a more structured sequence, followed by a clearer and more focused justification for the use of GEMS-9.

**3) More importantly, flesh out the motivation for using GEMS second-order factors.** Provide a stronger rationale for collapsing GEMS-9 to three second-order factors and report psychometric information, where available (internal reliabilities, inter-factor correlations…).

We agree that the rationale required further clarification. We have expanded the discussion of why the GEMS-9 second-order factors were selected, including their theoretical relevance and suitability for capturing music-specific emotional responses in large-scale designs. We now also report inter-factor correlations to increase transparency regarding the psychometric properties of the scales in our sample.

**4) The sentence on emotional differences “emerging accidentally during the 14th–16th centuries”** is ambiguous or unclear. Reword for clarity and cite an appropriate historical or psychoacoustic source (e.g., Parncutt, 2024).

Thank you for pointing this out. The sentence has been reworded for clarity, and we have added an appropriate reference to support the historical and psychoacoustic context.

**5) Replication vs. extension** – The study departs from previous methods and thus is not a strict replication. Please revise phrasing to “conceptual replication” or “extension,” and clearly distinguish replicated versus novel aspects.

We agree with this distinction. The study should indeed be described as a conceptual replication rather than a strict methodological replication. We have revised the terminology throughout the manuscript to clearly distinguish between replicated and novel aspects of the design.

**6) Unclear terminology: “pure major” and “pure minor”**

Thank you for noting this ambiguity. The terms “pure major” and “pure minor” have now been removed for clarity. The intention was simply to indicate that the stimuli were constructed in major and minor modes without modal mixture. As this terminology was potentially confusing and not essential, we have simplified the wording accordingly.

**7) I understand the rationale behind using control stimuli, but couldn’t one raise the issue that control stimuli are auditory as well?**

This is an important point. While alternative control modalities (e.g., visual stimuli) could have been used, we chose auditory control stimuli to ensure modality consistency and to isolate musical structure rather than sensory modality effects. We have now clarified this rationale in the introduction and explicitly addressed the limitations of this choice.

**8) Describe the translation procedure (forward/back translation, expert review) and report internal consistency statistics.**

We conducted forward and back translation procedures to ensure linguistic accuracy. Although an external expert review was not performed at the time, the translated instrument has since been used in multiple studies without issues. Unfortunately, internal consistency statistics were not computed in the original dataset; we acknowledge this as a limitation and clarify the translation procedure more explicitly in the manuscript.

**9) Specify the hosting platform (e.g., Qualtrics, Gorilla, PsychoPy online), playback format, use of headphones...**

We have added information about the hosting platform and relevant technical details in the Materials section. We previously mentioned headphone use in the limitations section; this has now also been clarified in the Procedure section for greater transparency.

**10) Averaging all five stimuli into single scores affects stimulus variability. Please test for composition effects** (e.g., mixed-effects model with composition as a random factor) or at least report variability across compositions to demonstrate generalizability.

Thank you for this important methodological point. We have now included additional analyses addressing potential composition effects. These results are reported in the Supplementary Materials, and we reference them explicitly in the Analysis and Results sections to demonstrate generalizability across stimuli.

**11) Add recent and relevant sources on major-minor perception, such as: Carraturo, G. et al. (2024).** The major–minor mode dichotomy in music perception. Physics of Life Reviews.Parncutt, R. (2024). Psychoacoustic Foundations of Major–Minor Tonality. MIT Press.

Thank you for these helpful suggestions. These publications were not yet available when the manuscript was originally submitted. We have now incorporated them where relevant to strengthen the theoretical framing of major–minor perception.
